# Prospective Multicenter International Surveillance of Azole Resistance in *Aspergillus fumigatus*

**DOI:** 10.3201/eid2106.140717

**Published:** 2015-06

**Authors:** J.W.M. van der Linden, M.C. Arendrup, A. Warris, K. Lagrou, H. Pelloux, P.M. Hauser, E. Chryssanthou, E. Mellado, S.E. Kidd, A.M. Tortorano, E. Dannaoui, P. Gaustad, J.W. Baddley, A. Uekötter, C. Lass-Flörl, N. Klimko, C.B. Moore, D.W. Denning, A.C. Pasqualotto, C. Kibbler, S. Arikan-Akdagli, D. Andes, J. Meletiadis, L. Naumiuk, M. Nucci, W.J.G. Melchers, P.E. Verweij

**Affiliations:** Author affiliations: Radboud University Medical Centre Nijmegen, Nijmegen, the Netherlands (J.W.M. van der Linden, W.J.G. Melchers, P.E. Verweij);; Statens Serum Institute, Copenhagen, Denmark (M.C. Arendrup);; University of Aberdeen, Aberdeen, UK (A. Warris);; Catholic University Leuven, Leuven, Belgium (K. Lagrou);; Centre Hospitalier Universitaire, Grenoble, France (H. Pelloux);; Lausanne University Hospital and University of Lausanne, Lausanne, Switzerland (P.M. Hauser);; Karolinska University Hospital Solna, Stockholm, Sweden (E. Chryssanthou);; Instituto de Salud Carlos III, Madrid, Spain (E. Mellado);; The Alfred Hospital, Melbourne, Victoria, Australia (S.E. Kidd);; Università degli Studi di Milano, Milan, Italy (A.M. Tortorano);; Hopital Européen G. Pompidou and Université Paris 5, Paris, France (E. Dannaoui);; Oslo University Hospital and Rikshospitalet, Oslo, Norway (P. Gaustad);; University of Alabama at Birmingham, Birmingham, Alabama, USA (J.W. Baddley);; University of Münster, Münster, Germany (A. Uekötter);; University of Innsbruck, Innsbruck, Austria (C. Lass-Flörl);; North Western State Medical University, Saint Petersburg, Russia (N. Klimko);; University of Manchester and Mycology Reference Centre of the University Hospital of South Manchester, Manchester, UK (C.B. Moore, D.W. Denning);; Santa Casa de Misericordia de Porto Alegre and Universidade Federal de Ciencias da Saude de Porto Alegre, Porto Alegre, Brazil (A.C. Pasqualotto);; Royal Free London NHS Foundation Trust and University College London, London, UK (C. Kibbler);; Hacettepe University Medical School, Ankara, Turkey (S. Arikan-Akdagli);; University of Wisconsin, Madison, Wisconsin, USA (D. Andes);; Attikon University Hospital, Athens, Greece (J. Meletiadis);; Hospital of the Medical University of Gdansk, Gdansk, Poland (L. Naumiuk);; Universidade Federal do Rio de Janeiro, Rio de Janeiro, Brazil (M. Nucci)

**Keywords:** Aspergillus, azole resistance, aspergillosis, prevalence, international, fungi, surveillance, antimicrobial resistance

## Abstract

To investigate azole resistance in clinical *Aspergillus* isolates, we conducted prospective multicenter international surveillance. A total of 3,788 *Aspergillus* isolates were screened in 22 centers from 19 countries. Azole-resistant *A. fumigatus* was more frequently found (3.2% prevalence) than previously acknowledged, causing resistant invasive and noninvasive aspergillosis and severely compromising clinical use of azoles.

Azole resistance is increasingly recognized as a problem in aspergillus diseases (*1*). Within the *Aspergillus fumigatus* species complex, new sibling species have been reported to cause invasive aspergillosis; these species are generally intrinsically less susceptible than *A. fumigatus* sensu strictu to azole compounds (*2*). Acquired resistance to azoles in *A. fumigatus* has become a public health concern because of the presumed fungicide-driven route of resistance selection and the associated risk for geographic migration. Surveillance studies show that, in areas to which *Aspergillus* is endemic, the environmental route of resistance selection contributes to >90% of resistance mechanisms in azole-resistant aspergillus diseases (*1*,*3*). Azole resistance has been observed in patients with no recent history of azole therapy, and the mortality rate for patients with azole-resistant invasive aspergillosis was 88% (*3*).

## The Study

**Figure 1:**
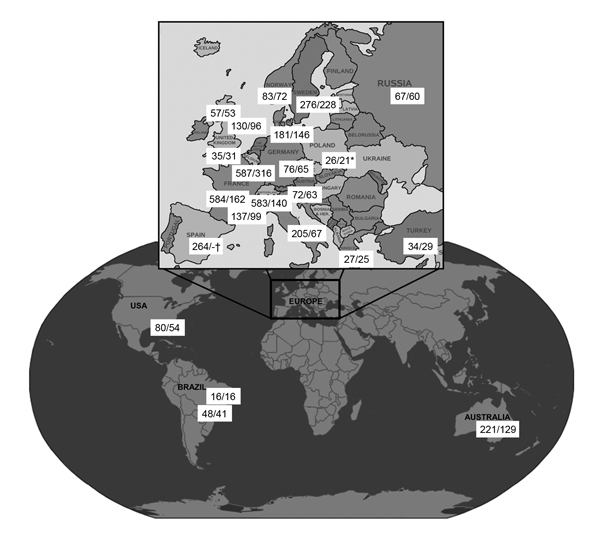
Study characteristics (number of isolates/patients screened) from 22 centers in 19 countries participating in a study of azole resistance in *Aspergillus fumigatus*. *Period screened was 8 months instead of 1 year in this center for unknown reason. †Total number of screened patients is unknown because this center is a reference laboratory that does not have access to patient characteristics.

Our objective was to investigate the prevalence of azole resistance in clinical *Aspergillus* isolates. A multicenter international surveillance network was established (Surveillance Collaboration on *Aspergillus* Resistance in Europe [SCARE-network]), comprising 22 centers from 19 countries (18 European and 4 non-European sites) (Figure 1). To detect azole-resistant *A. fumigatus*, we developed a phenotypic screening-method using a 4-well plate format with agar supplemented with itraconazole, voriconazole, and posaconazole (*4*). Each center was asked to screen for azole resistance for 12 consecutive months. For each screened isolate, patient characteristics were registered through an online questionnaire, and patients with invasive aspergillosis were classified according to the European Organization for the Research and Treatment of Cancer/Mycoses Study Group consensus definitions (*5*).

For every *A. fumigatus* isolate that grew on any of the azole-containing wells, the primary culture isolate was sent both to the Radboud University Medical Centre (Nijmegen, the Netherlands) and Statens Serum Institute (Copenhagen, Denmark) for molecular species identification, susceptibility testing according to the EUCAST (European Committee on Antimicrobial Susceptibility Testing) broth microdilution reference method (*6*), and determination of the full coding sequence of both strands of the *cyp51A* gene and the promoter region by PCR amplification. For every resistant isolate, a susceptible control isolate was assigned; this control isolate was the first susceptible isolate screened on the 4-well plate format in the same center after the resistant isolate, and they received molecular species identification and susceptibility testing according to the EUCAST broth microdilution reference method (*6*).

**Figure 2:**
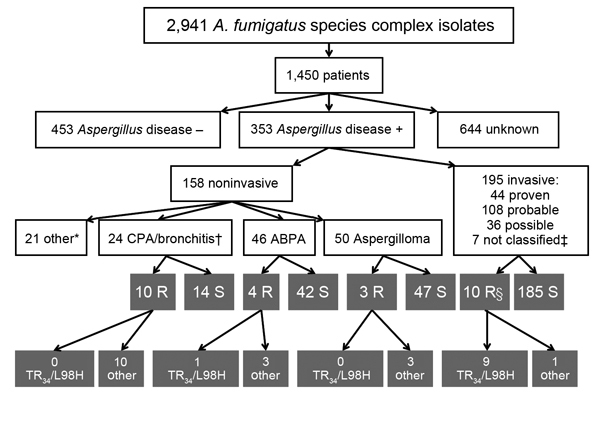
Patient characteristics and underlying resistance mechanisms of patients with invasive and noninvasive *Aspergillus* disease. *Otomycosis, dermatomycosis, or onychomycosis; 1 patient had a resistant isolate and otomycosis (patient 9 in the online Technical Appendix Table, http://wwwnc.cdc.gov/EID/article/21/6/14-0717-Techapp1.pdf). †One patient had chronic pulmonary aspergillosis and ABPA. ‡Not classified according to European Organization for the Research and Treatment of Cancer/Mycoses Study Group criteria (*5*). §One patient is included with 46-bp tandem-repeat resistance mechanism. ABPA, allergic bronchopulmonary aspergillosis; CPA, chronic pulmonary aspergillosis; R, resistant; S, susceptible; –, negative; +, positive.

During January 2009–January 2011, a total of 3,788 *Aspergillus* isolates were screened for azole resistance by using the 4-well plates (Figure 1). Clinical information was available from 1,911 patients from 21 centers in 18 countries. Most (2,941 [77.6%]) isolates were classified as *A. fumigatus* species complex and were recovered from 1,450 patients. The most common underlying disease was chronic lung disease (30.0%), followed by cystic fibrosis (22.1%) and hemato-oncologic diseases (12.9%). A total of 204 (14.1%) patients had undergone hematopoietic stem cell transplantation or solid organ transplantation, and 265 (18.3%) had been treated with corticosteroids within 3 months before culture of the isolate. A total of 223 (15.4%) of 1,450 patients had received antifungal drugs within 3 months before, or at the time of the positive culture. For 806 (55.6%) patients, the clinical relevance of the cultured *A. fumigatus* sc isolate was reported (Figure 2).

For 60 *A. fumigatus* species complex isolates, the resistant phenotype was confirmed in vitro (Table 1). Forty-seven (78.3%) azole-resistant isolates were identified as *A. fumigatus* sensu strictu. The other 13 azole-resistant isolates were identified as *A. lentulus* (7 isolates), *Neosartorya pseudofisheri* (4 isolates), and *N. udagawae* (2 isolates). Sequence analysis of the *cyp51A* gene of *A. fumigatus* showed TR_34_/L98H in 23 (48.9%) azole-resistant *A. fumigatus* isolates (Table 2). All TR_34_/L98H isolates were cultured from patients from European centers. In 3 isolates from the Netherlands, the TR_46_/Y121F/T289A resistance mechanism was found (*7*).

**Table 1:** Susceptibility for 3 antifungal medical azoles and 1 azole fungicide of resistant isolates (60 isolates) and control group (60 isolates) after species identification

Isolate	Median MIC, mg/L			
	Itraconazole	Voriconazole	Posaconazole	Tebuconazole
Azole-resistant *Aspergillus fumigatus*, n = 47	>8	2	1	8
*A. fumigatus* sibling species,* n = 13	1	2	0.25	>8
*A. fumigatus* controls, n = 60	0.25	0.5	0.06	2

*Aspergillus lentulus, *Neosartorya pseudofischeri*, *N. udagawae*.

**Table 2:** Acquired resistance mechanisms from each country in c*yp51A* gene in 47 *Aspergillus fumigatus* isolates with an azole-resistant phenotype

Country	No. azole-resistant isolates, n = 47	TR_34_/L98H or TR_46_/Y121F/T289A mechanism (no. isolates)	Other mutations (no. isolates)	No. isolates without Cyp51A-mutations
Austria	2	TR_34_/L98H (2)	0	0
Belgium	8	TR_34_/L98H (7)	F46Y/M172G (1)	0
Denmark	6	TR_34_/L98H (4)	0	2
France	4	TR_34_/L98H (1)	G54W (1)	2
Italy	5	TR_34_/L98H (5)	0	0
The Netherlands	7	TR_34_/L98H (4), TR_46_/Y121F/T289A (3)	0	0
Spain	1	No isolates	0	1
Sweden	1	No isolates	F46Y/M172G	0
United Kingdom	13	No isolates	P381R/D481E (1), L329V (1), M220K (1), L77V/L399I/D481E (1), M220I (3), M220R (1), G54R (1), G54E (1), G54W (1)	2
Resistant isolates, %	100	55.3	29.8	14.9

A total of 60 azole-resistant isolates were recovered from 46 patients. The overall prevalence of azole resistance among patients with *A. fumigatus* species complex isolates was 3.2% (range 0.0%–26.1% among the centers). Azole resistance was detected in 11 (57.9%) of 19 participating countries. Acquired resistance in *A. fumigatus* was found at European sites: Austria, Belgium, Denmark, France, Italy, the Netherlands, Spain, Sweden, and the United Kingdom. In 5 countries (Australia, Germany, Spain, Sweden, and United Kingdom), azole-resistant *A. fumigatus* sibling species were recovered.

From the 46 patients with resistant isolates, 8 patients had azole-resistant *A. fumigatus* sibling isolates, and 38 had a resistant *A. fumigatus* isolate. Of these 38 patients, 19 had an isolate that harbored a fungicide-driven resistance mechanism (i.e., TR_34_/L98H or TR_46_/Y121F/T289A). When comparing patients with isolates harboring presumed fungicide-driven resistance mechanisms with “non–fungicide driven” resistance mechanisms (i.e., point mutations or non–*Cyp51A*-mediated mechanisms), azole exposure differed significantly: 4 (21.1%) of 19 patients with an isolate with the fungicide-driven resistance mechanism had a history of azole therapy, compared with 16 (84.2%) of 19 patients with isolates with other or no *cyp51A* mutations (p = 0.001). Of the 195 cases with invasive aspergillosis, azole resistance was documented in 10 (5.1%) (3 proven, 1 probable, and 6 possible infections). Among the patients with resistant isolates, 28 patients had documented aspergillus disease (Technical Appendix Table). The case-fatality rate for this cohort was 70%.

## Conclusions

Acquired azole resistance in *A. fumigatus* was detected in 11 of 17 European centers in 9 countries. Overall prevalence of azole resistance was 3.2%; TR_34_/L98H was the predominant mechanism of resistance (48.9%) in *A. fumigatus* sensu strictu isolates. This finding substantiates our concern that azole resistance is an emerging problem in *A. fumigatus* and that resistance selection in the environment contributes significantly to azole-resistant aspergillus diseases. A predilection of isolates harbored the TR_34_/L98H mutation for patients with acute invasive diseases over patients with aspergilloma and chronic pulmonary aspergillosis. Azole-resistant invasive aspergillosis was documented in 5.1% of cases of invasive aspergillosis, which is not lower than the percentage of the prevalence of azole resistance among the *A. fumigatus* isolates (3.2%). This finding might indicate that resistance does not come with a significant fitness cost and that azole-resistant isolates that harbored TR_34_/L98H or TR_46_/Y121F/T289A are at least as capable of causing invasive aspergillosis as nonresistant wild-type isolates. Although the clinical implications of sibling species of *A. fumigatus* are less well understood, our study confirms that these species are generally less susceptible than *A. fumigatus* to azole antifungal drugs.

Our study shows that azole resistance is widespread in Europe. Azole-resistant *A. fumigatus* caused aspergillus diseases in the patients in our study, and azole resistance was associated with a worsened outcome (*3*). A rapid and convenient screening method for resistance is indispensable, and centers that care for patients with aspergillus diseases should perform surveillance to determine their local epidemiology. Furthermore, azole resistance has become a public health problem that needs continued international surveillance and research on the mechanisms that enable its selection in the environment. This report of the emergence of resistance has launched a new phase in the management of aspergillus diseases. Unless we can implement measures that prevent the fungicide-driven route of resistance development, the clinical use of azoles will be severely compromised.

**Technical Appendix.** Clinical characteristics of 28 patients with documented azole-resistant *Aspergillus* diseases.
