# Supplementary material for: Prospective Multicenter International Surveillance of Azole Resistance in Aspergillus fumigatus
Source: Emerg Infect Dis. 2015 Jun;21(6):1041–4. doi: 10.3201/eid2106.140717 (PMC4451897; doi:10.3201/eid2106.140717)
Supplement: Supplementary file 1 — Technical Appendix. Clinical characteristics of 28 patients with documented azole-resistant Aspergillus diseases. [file 14-0717-Techapp-s1.pdf]

Article DOI: http://dx.doi.org/10.3201/eid2106.140717

Prospective Multicenter International Surveillance of Azole Resistance in *Aspergillus fumigatus*

Technical Appendix Table. Clinical characteristics of 28 patients with documented azole-resistant *Aspergillus* diseases*

Patient no.	Sex/ age, y	Country	Underlying condition	Corticosteroid use	Date, specimen	Species (no. isolates)	Resistance mechanism	*Aspergillus* disease	Previous treatment	Outcome
1	F/29	Denmark	Cystic fibrosis	No	2009 May, sputum	*A. fumigatus* (2)	TR_{34}/L98H	ABPA	VCZ	Survived
2	M/75	Italy	Pulmonary disease	No	2009 May, sputum	*A. fumigatus*	TR_{34}/L98H	Possible IA	None	Survived
3	M/66	Netherlands	Hematologic disease	Yes	2009 May, sputum	*A. fumigatus*	TR_{34}/L98H	Possible IA	None	Died
4	F/61	UK	Pulmonary disease	Yes	2009 May, sputum	*A. fumigatus*	M220K, E317G	CPA/ABPA	AMB	Died
5	M/55	UK	Oncologic disease	No	2009 May, sputum	*A. fumigatus*	L329V	CPA	ITZ	Died
6	F/18	UK	Cystic fibrosis	No	2009 May, sputum	*A. fumigatus*	P381R, D481E	Bilateral Aspergilloma	VCZ	Died
7	F/62	Netherlands	Hematologic malignancy, HSCT	Yes	2009 Aug, mouth wash	*A. fumigatus*	TR_{34}/L98H	Possible IA	None	Survived
8	M/61	Netherlands	Hematologic malignancy, HSCT	Yes	2009 Aug, sputum	*A. fumigatus*	TR_{34}/L98H	Possible IA	None	Died
9	F/30	Austria	Otomycosis	No	2009 Sep, ear swab sample	*A. fumigatus* (2)	TR_{34}/L98H	Otomycosis	None	Survived
10	M/60	Netherlands	Hematologic malignancy, HSCT	Yes	2009 Sep, sputum	*A. fumigatus*	TR_{34}/L98H	Possible IA	None	Survived
11	F/50	UK	Pulmonary disease	No	2009 Sep, sputum	*A. fumigatus*	L77V, L399I, D481E	CPA	VCZ	Ongoing infection
12	M/16	Denmark	Cystic fibrosis	No	2009 Oct, sputum	*A. fumigatus*	No Cyp51A mutation	ABPA	VCZ	Survived
13	M/71	UK	Pulmonary disease	No	2009 Oct, sputum	*A. fumigatus*	M220I, L319V	CPA	POS	Died
14	M/57	UK	Pulmonary disease	No	2009 Oct, sputum	*A. fumigatus*	No Cyp51A mutation	CPA	AMB	Ongoing infection
15	F/56	UK	Pulmonary disease	No	2009 Nov, sputum	*A. fumigatus*	G54R	CPA	POS	Ongoing infection
16	M/80	UK	Pulmonary disease	No	2009 Nov, sputum	*A. fumigatus*	M220R	CPA	ITZ	Died
17	M/19	France	Cystic fibrosis	No	2009 Dec, sputum	*A. fumigatus*	No Cyp51A mutation	Bronchitis	VCZ	Ongoing infection
18	F/59	UK	Pulmonary disease	No	2009 Dec, sputum	*A. fumigatus*	No Cyp51A mutation	Aspergilloma	VCZ	Died
19	M/75	Belgium	Pulmonary disease	Yes	2010 Jan, BAL	*A. fumigatus*	TR_{34}/L98H	Probable IA	None	Died
20	F/51	Netherlands	Kidney transplant	Yes	2010 Jan, kidney biopsy sample	*A. fumigatus* (3)	TR_{46}/Y121F/T289A	Proven IA	None	Died
21	M/67	UK	Pulmonary disease	No	2010 Jan, sputum	*A. fumigatus*	G54E	CPA	POS	Ongoing infection

Patient no.	Sex/ age, y	Country	Underlying condition	Corticosteroid use	Date, specimen	Species (no. isolates)	Resistance mechanism	*Aspergillus* disease	Previous treatment	Outcome
22	M/54	Denmark	Cystic fibrosis, lung transplant	No	2010 Jan, BAL	*A. fumigatus* (2)	TR_{34}/L98H	Possible IA	VCZ	Died
23	M/62	Belgium	Pulmonary disease, lung transplant	Yes	2010 Feb, lung biopsy sample	*A. fumigatus*	TR_{34}/L98H	Proven IA	VCZ, POS	Died
24	F/26	Australia	Cystic fibrosis	Yes	2010 Feb, sputum	*N. pseudofischeri*	Unknown	ABPA	VCZ	Survived
25	M/78	UK	Pulmonary disease	No	2010 Mar, sputum	*A. fumigatus*	G54W, Q249H	CPA	POS	Died
26	M/41	UK	Cystic fibrosis	Yes	2010 Mar, sputum	*N. pseudofischeri*	Unknown	ABPA	AMB	Ongoing infection
27	M/53	Denmark	Pulmonary disease	No	2010 Apr, sputum	*A. fumigatus*	No Cyp51A mutation	Aspergilloma	ITZ	Survived
28	M/71	UK	Pulmonary disease	No	2010 May, sputum	*A. fumigatus*	M220I	Proven IA	AMB, VCZ, POS	Died

*ABPA, allergic bronchopulmonary aspergillosis; IA, invasive aspergillosis; AMB, amphotericin B; BAL, bronchoalveolar lavage fluid; CPA, chronic pulmonary aspergillosis; HSCT, hematopoietic stem cell transplantation; ITZ, itraconazole; POS, posaconazole; VCZ, voriconazole.
